# Proteome and physiological analyses reveal tobacco (*Nicotiana tabacum*) peroxidase 7 (POD 7) functions in responses to copper stress

**DOI:** 10.1007/s11248-022-00310-0

**Published:** 2022-07-06

**Authors:** Qian Gao, Li Xu, Xiang Li, Wenwu Yang, Qili Mi, Liming Lu, Xin Liu, Kai Wang, Yifei Lu, Zhangyu Chen, Xuemei Li, Liqin Li

**Affiliations:** 1 Yunnan Key Laboratory of Tobacco Chemistry, R&D Center of China Tobacco Yunnan Industrial Co. Ltd., Kunming, 650202 Yunnan People’s Republic of China; 2 Agriculture College, Sichuan Agriculture University, Chengdu, 611130 Sichuan People’s Republic of China

**Keywords:** Copper, *Nicotiana tabacum*, Proteomics, Peroxidase7, Stress

## Abstract

Copper is a micronutrient essential for plant growth and development. However, Cu is also a heavy metal element that has deleterious impacts on plants when excessively accumulated in the environment. To understand the molecular mechanism underlying tobacco in response to Cu stress, iTRAQ based technology was used to identify differentially expressed proteins (DEPs) and important metabolic pathways in tobacco plants treated with excessive CuSO4. The results showed that 180 DEPs were detected between the treatment and control, among which 78 were upregulated and 102 were downregulated. These DEPs can be functionally divided into 65 categories and are closely related to metabolic pathways, carbon metabolism, secondary metabolite biosynthesis, biosynthesis of antibiotics, glyoxylate and dicarboxylate metabolism, and glycolysis/gluconeogenesis. Peroxidase7 was significantly upregulated and was selected and overexpressed in tobacco. Then, positive transgenic lines and wild type plants were exposed to a Cu stress environment. The results showed that Peroxidase7 transgenic tobacco plants exhibited enhanced Cu stress resistance with decreased malondialdehyde and Cu contents, and increased shoot dry weight, root length, secondary root number, SOD, POD and CAT activity. The present study suggests that the ROS scavenging mechanism is essential for tobacco plants in response to Cu stress and that Peroxidase7 functions in tobacco plant resistance to excessive Cu environment.

**Supplementary Information:**

The online version contains supplementary material available at 10.1007/s11248-022-00310-0.

## Introduction

In higher plants, as a structural and catalytic component of many important proteins, copper (Cu) is a basic mineral element required for plant growth and development. Cu is a structural component of Cu–Zn superoxide dismutase, cytochrome oxidase, phenol oxidase, ascorbic acid oxidase, polyamine oxidase, plastocyanin and ETR and participates in many metabolic and physiological processes, such as photosynthesis and respiration, the metabolism of nitrogen compounds and carbohydrates, amino acid activation and protein biosynthesis promotion, signal transduction, cell wall and flower organ formation, and stress response (Burkhead et al. 2009; Marschner 1995). However, Cu is also one of the heavy metal elements that have adverse impacts on plants when excessively accumulated in the environment. These negative effects include the inhibition of many essential physiological and biochemical processes, such as photosynthesis and root development, leading to the growth retardation (Sudo et al. 2008; Gajewska et al. 2010; Nair and Chung 2014; Chrysargyris et al. 2019).

Much evidence indicates that environmental factors including Cu, can generate ROS (reactive oxygen species) in plant cells (Huang et al. 2019). On one hand, ROS can trigger a signal transduction cascade in the cell nucleus as a signal molecule, and on the other cause cellular damage leading to plant morphological structure alterations by their strong oxidative properties (Bose et al. 2014). ROS can regulate signaling and affect the protein redox status through the oxidation of thiol groups of cysteines and methionine residues. These redox-related processes are firmly modulated by proteins such as thio- and glutaredoxins (Waszczak et al. 2018). Recently, a new redox-based sensing mechanism for H_2_O_2_ was reported, which links extracellularly generated ROS, cell-surface H_2_O_2_ receptors, and intracellular signaling cascades (Dvorak et al. 2021). Through retrograde signaling, redox perturbations caused by ROS generated in mitochondria and chloroplasts are transduced to activate defense gene expression in nucleus (Chan et al. 2016). H_2_O_2_ produced in plastids can be transported to the nucleus to activate adaptive mechanisms by retrograde signaling during which ROS act as mediators (Exposito-Rodriguez et al. 2017).

To survive in a Cu stressed environment, plants have developed delicate mechanisms to deal with Cu stress at physiological level such as exclusion and compartmentalization, and/or the molecular level by regulating gene expression, protein synthesis and activity (Song et al. 2013). Among them, the antioxidant defense system mediated ROS scavenging mechanism is essential for the plant response to Cu-induced oxidative stress. These systems include enzymatic and nonenzymatic components, such as ascorbate peroxidase (APX), peroxidase (POD), catalase (CAT), superoxide dismutase (SOD), glutathione reductase (GR), dehydroascorbate reductase (DHAR), glutathione (GSH) and ascorbic acid (AsA).

Several studies have revealed the roles of antioxidants played in the response of plants to Cu stress. In rice, trehalose pretreatment resulted in the enhanced activities of major antioxidant enzymes leading to lower ROS accumulation in rice seedlings exposed to excessive Cu, and eventually improved Cu tolerance in rice plants (Mostofa et al. 2015). Two transgenic tobacco lines carrying the *AtTPS1* (trehalose-6-phosphate synthase) gene from Arabidopsis exhibited increased catalase activity and better acclimation to excess Cu compared to WT (Martins et al. 2014).

In grapevine, most ROS detoxification systems, including antioxidant enzymes, such as APX, POD, CAT and GST (glutathione S-transferase), were strongly upregulated under Cu stress (Leng et al. 2015) indicating the ROS eliminating isoforms may be vital in the physiological response to Cu stress. According to Hamed et al. (2017), increased activities of GST, APX, GR and SOD enzymes combined with elevated glutathione levels and other stress-resistance substances, such as proline, polyphenols, flavonoids and tocopherols, contributed to the lower Cu stress sensitivity of the green microalgae *Chlorella sorokiniana*. Proteome analysis conducted by Song et al. (2013) also showed that significant upregulation of POD, L-ascorbate peroxidase and DHAR was observed in Cu-treated rice. Thus, *Colobanthus quitensis* exhibits tolerance to Cu by recruiting antioxidant defensive systems involving nonenzymatic antioxidants such as phenolics, GSH and ascorbic acid (Contreras et al. 2018). *Spirodela polyrhiza* has the ability to resist Cu toxicity by upregulating of antioxidant enzymes and proline content (Singh et al. 2020). Ascobin (ascorbic acid: citric acid at 2:1) and glutathione pretreatments exhibited less inhibitory effects of Cu stress on rice plants by improving antioxidant enzyme activities and decreasing levels of ROS and malondialdehyde (MDA) (Tahjib-Ul-Arif et al. 2021).

Proteomics is a powerful tool in the investigation of the molecular mechanisms of plants in response to heavy metal stresses (Ahsan et al. 2010; Li et al. 2013; Song et al. 2013; Roy et al. 2016; Gong et al. 2017). Among proteomics analysis methods, iTRAQ (isobaric tags for relative and absolute quantification) plays a vital role in quantitatively measuring plant proteome changes under abiotic stresses, and is therefore widely used in exploring the mechanisms of plant heavy metal resistance. For instance, in order to understand plant proteome changes in response to aluminum (Al) stress, iTRAQ based proteome analysis was employed in rice (Wang et al. 2014), sorghum (Zhou et al. 2017) and duckweed (Su et al. 2019), and some important proteins involved in antioxidant enzymes were identified, providing useful information underlying plant Al resistance.

Tobacco is an important crop widely cultivated across the world. In tobacco cultivation practice, unfavorable environments, such as biotic and abiotic stresses, including heavy metal stress, are often encountered, leading to huge yield losses. Therefore, understanding the molecular mechanisms underlying tobacco stress tolerance is vital for the sustainable development of tobacco cultivation. However, the molecular mechanism underlying this response remains unclear. Thus, we performed a physiological experiment, in which tobacco seedlings were treated with excessive CuSO4, and identified differentially expressed proteins (DEPs) and important metabolic pathways with iTRAQ based technology. Then, tobacco *peroxidase* 7 was cloned and overexpressed in common tobacco (*Nicotiana tabacum*, *ver* K326). The phenotypes of the WT and transgenic lines were screened under Cu stress, and several important physiological indices were measured. The hypothesis is that excessive Cu application can alter protein expression profile and change the behavior of physiological and biochemical processes in tobacco plants, and POD plays essential roles in the tobacco response to Cu stress. Our study convincingly demonstrates an association of protein variations in tobacco plants subjected to Cu stress, and that *peroxidase* 7 functions in the tobacco response to Cu stress.

## Materials and methods

### Plant growth and Cu stress treatment

Seeds of tobacco cultivar K326 (*Nicotiana tabacum ver* K326), obtained from Yunnan Key Laboratory of Tobacco Chemistry, were surface-disinfected with 15% NaClO, sowed on Murashige-Skoog (MS) medium (Murashige and Skoog 1962) and grown for 6 weeks in a greenhouse. The temperature in the greenhouse was 24 °C with 16 h of light and 8 h of darkness.

After this period, tobacco plants were divided into two groups: control and Cu stress treatments. Each group contained three replicates with 15 plants for each replicate. Tobacco plants from the control group were continually cultivated in the same MS medium without any additional substance. For Cu stress treatment, the MS medium for tobacco plant growth was supplemented with 5 mg per ml copper sulfate. Seven days after treatment, tobacco plants from both groups were harvested for protein profile analysis.

For physiological parameter detection of tobacco plants from wild-type and transgenic lines, tobacco plants were grown for eight weeks in the same greenhouse, classified into control and Cu-treated groups, and treated with 2.5 mg/ml copper sulfate and water, respectively. One week after this treatment, tobacco plants from each group were collected, and physiological index detection was performed.

### Protein preparation, digestion, iTRAQ labeling and fractionation

Seven days after treatment, plant leaves were collected from the control and Cu-stressed tobacco plants. The crude protein was prepared for iTRAQ analysis under the guidance of Wang et al. (2015).

Protein concentrations from plant samples were measured by the Bradford assay. Protein alkylate digestion was conducted as described by Li et al. (2019). Next, the iTRAQ Reagent-8 plex Multiplex Kit (AB Sciex U.K.) was employed to label protein samples following the manufacturer's instructions. Then, the labeled proteins were fractionated with an HPLC system, and the collected fractions were used for nano-LC–MS/MS analysis.

### Protein identification and data analysis

An AB SCIEX nano-LC–MS/MS system was employed to perform protein identification. The yielded raw MS/MS file data were transferred to ProteinPilot software v4.0 for protein analysis against the UniProt Poaceae database. During this process, the criteria of coverage more than 5% and/or more than two peptides identified were needed.

Protein abundance was determined by means of the median abundance in Mascot. The fold change in protein expression indicates the protein abundance ratio to be compared between samples. A significantly differentially expressed protein (DEP) refers to a protein with a fold change greater than 1.5 (treatment compared to CK ratio 1.5), or less than 0.66 (treatment compared to CK ratio 0.66) and a *P* value less than 0.05.

To decrease false-positive rate, Peng et al.’s (2003) method was used. Briefly, three parameters were utilized including Δ*Cn* (delta-correlation score), *Xcorr* (Sequest cross-correlation score) and state of tryptic ends (fully- or partially tryptic). Δ*Cn* was larger than 0.08. For charge states of + 1, + 2, + 3, fully tryptic peptides must have *Xcorr* score greater than 2.0, 1.5, or 3.3, respectively. In contrast, in the case of partially tryptic peptides, for charge states of + 2 or + 3, *X*corr score was more than 3.0 or 4.0 respectively. In our study, minimum score for peptides was set > 40, and the false-positive rate of the DEPs was adjusted to < 1%.

### Bioinformatics analysis of the differentially expressed proteins (DEPs)

To determine the functional characteristics of the identified DEPs, Blast2GO v3.0 software was used to map these proteins with Gene Ontology Terms (http://geneontology.org/). Next, WEGO software (http://wego.genomics.org.cn) was employed to perform GO classification of the identified DEPs. Then, Kyoto Encyclopedia of Genes and Genomes (KEGG) pathway analysis of these proteins was conducted by using KOBAS 3.0 software (http://kobas.cbi.pku.edu.cn/help.do). Finally, STRING v10.5 software (http://string-db.org) was used to construct the interaction network of the identified DEPs.

### qRT-PCR analysis

Twelve proteins were randomly selected for qRT-PCR analysis in order to validate the results of protein expression levels determined by the iTRAQ method. Of the 12 genes, eight genes were selected from upregulated DEPs, namely *APX*, *Peroxidase*, *GST*, *Catalase2*, *USP* (universal stress protein), *PR4* (pathogenesis-related 4), *HSP20* (heat shock protein 20) and *G3PD* (glyceraldehyde-3-phosphate dehydrogenase); four genes were from downregulated DEPs, as *OEE* (oxygen-evolving enhancer protein 1 of photosystem II), *RLP* (LRR receptor-like serine_threonine-protein kinase), *CabBP* (chlorophyll A-B binding family protein) and *GS* (Glutamine synthetase). Among them, *APX*, *Peroxidase*, *Catalase2* and *GST* are from ROS scavenging system; *HSP20*, *PR4* and *USP* from stress-related proteins; *G3PD* from energy metabolic process; *RLP* from signal transduction process; *OEE* and *CabBP* from photosynthesis; *GS* from nitrogen metabolic process.

To do this, TRIzol reagent (Invitrogen, Carlsbad, USA) was used to prepare total RNA from plant samples collected from the Cu treatment and control groups seven days after Cu treatment following the manufacturer’s protocol. cDNA synthesis was conducted using a cDNA synthesis kit (Thermo, USA). In performing qRT-PCR, the internal control was the EF1αL (elongation factor 1 alpha-like) gene, and the 2(-delta delta C(T)) method (Livak and Schmittgen 2001) was used to calculate relative gene expression level.

### Cloning and genetic transformation of tobacco *peroxidase7*

The method of cloning and genetic transformation of tobacco *peroxidase7* was performed according to Lu et al. (2020). Primers for *peroxidase7* cloning were designed according to the CDS of *peroxidase7* (accession XP_016506030.1). A plant transformation plasmid (pBI121- *peroxidase7*) was constructed and the leaf disc cocultivation method was used for gene transformation. Genomic PCR was performed to select kanamycin-resistant positive transgenic tobacco plants. The specific primers used in this study for gene cloning and the selection of kanamycin-resistant positive transgenic tobacco plants are listed in Supplementary Table S1.

### Plant dry weight, root length and secondary root number measurement

Seven days after treatment, tobacco plants from the control and Cu stress treatments were collected, and the plant dry weight, the length of the main root and secondary root number were determined. For plant dry weight measurement, tobacco plants were divided into shoots and roots, and placed in an oven at 80 °C until constant weight was achieved, and analyzed.

### Malondialdehyde content measurement

Seven days after Cu treatment, plant leaves from Cu-treated and control plants were collected for the measurement of MDA content. Plant leaves (1.0 g) were mixed with 10 ml of 10% TCA (trichloroacetic acid) solution, ground thoroughly, and centrifuged at 5000 × g for 10 min to yield the crude MDA extract. Next, 0.6% TBA (thiobarbituric acid) (m/v) was added to 2.0 ml of supernatant and the mixture was transferred to a boiling pot for 15 min, cooled and centrifuged for 10 min at 5000 × g. Then, the absorbance of the supernatant was measured at 600 nm, 532 nm and 450 nm respectively, and the MDA content of plant leaves was calculated accordingly.

### Cu content determination

Cu contents in tobacco plant leaves were determined according to Martin et al. (2014). Briefly, tobacco plant samples were placed in an electric oven at 80 °C until dry and then ground into a fine powder. Samples (0.5 g) were prepared and digested for 45 min with 5 mL of concentrated nitric acid in a microwave oven. The Cu content was then analyzed with the resulting solution by atomic absorption spectrophotometry (Jena, Nov AA. 400).

### Determination of antioxidant enzyme activity

The peroxidase (type III) activity of tobacco plants from the control and Cu stress treatments was measured following the manufacturer’s protocols using a peroxidase measurement kit (Jiancheng Inc, China). Superoxide dismutase (SOD) activity was determined using the spectrophotometric method, according to Giannopolitis and Ries (1977). Catalase (CAT) activity was measured by the determination of the optical density of plant samples at 240 nm, according to Farhad et al. (2011).

### Statistical data analysis

For statistical data analysis, all data from this study were collected from three biological replicates. The significance between treatments was compared using the *F*-test and one way ANOVA at levels of *P* ≤ 0.01 and/or *P* ≤ 0.05. “*” and “**” represent significant differences at the *P* ≤ 0.05 and/or *P* ≤ 0.01 levels, respectively, among the samples in the figures. Excel and the SPSS 14.0 statistical software programs were employed for data analysis.

## Results

### Identification of DEPs in tobacco plants under Cu stress

Total proteins were extracted from plant samples of the control and Cu treatment groups as mentioned before and were applied for iTRAQ analysis. We identified a total of 3047 proteins from these plant samples. After the calculation of fold-changes of proteins by comparing Cu treatment to control, a total of 180 proteins showed significant differential expression, among them, 78 and 102 DEPs were significantly increased (the ratio of treatment to control ≥ 1.5) and decreased (the ratio of treatment to control ≤ 0.66), respectively (*P* < 0.05, Supplementary Table S2).

### Functional annotation of the DEP response to Cu stress

**Fig. 1:**
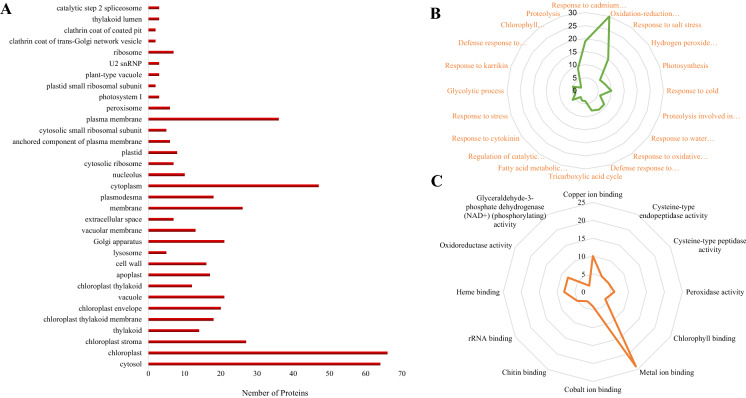
GO analysis of the DEPs of Cu-stressed tobacco plants. The functional annotation of all the DEPs was performed using WEGO (Web Gene Ontology Annotation Plot) software. **A** cellular component (CC) category. **B** molecular function (MF) category. C, biological processes (BP) category

Web Gene Ontology Annotation Plot (WEGO) software was used to conduct gene ontology analysis, and the result showed that all the DEPs functionally fell into three main categories as molecular function (MF), biological processes (BP) and cellular component (CC), and 65 subcategories. Among them, the most abundant DEPs were involved in the chloroplast (39.3%), cytosol (38.1%), oxidation–reduction process (17.9%), response to cadmium ion (11.3%), metal ion binding (14.3%) and copper ion binding (6.0%) (Fig. 1).

### Identification of metabolic processes that were significantly changed after Cu treatment

**Fig. 2:**
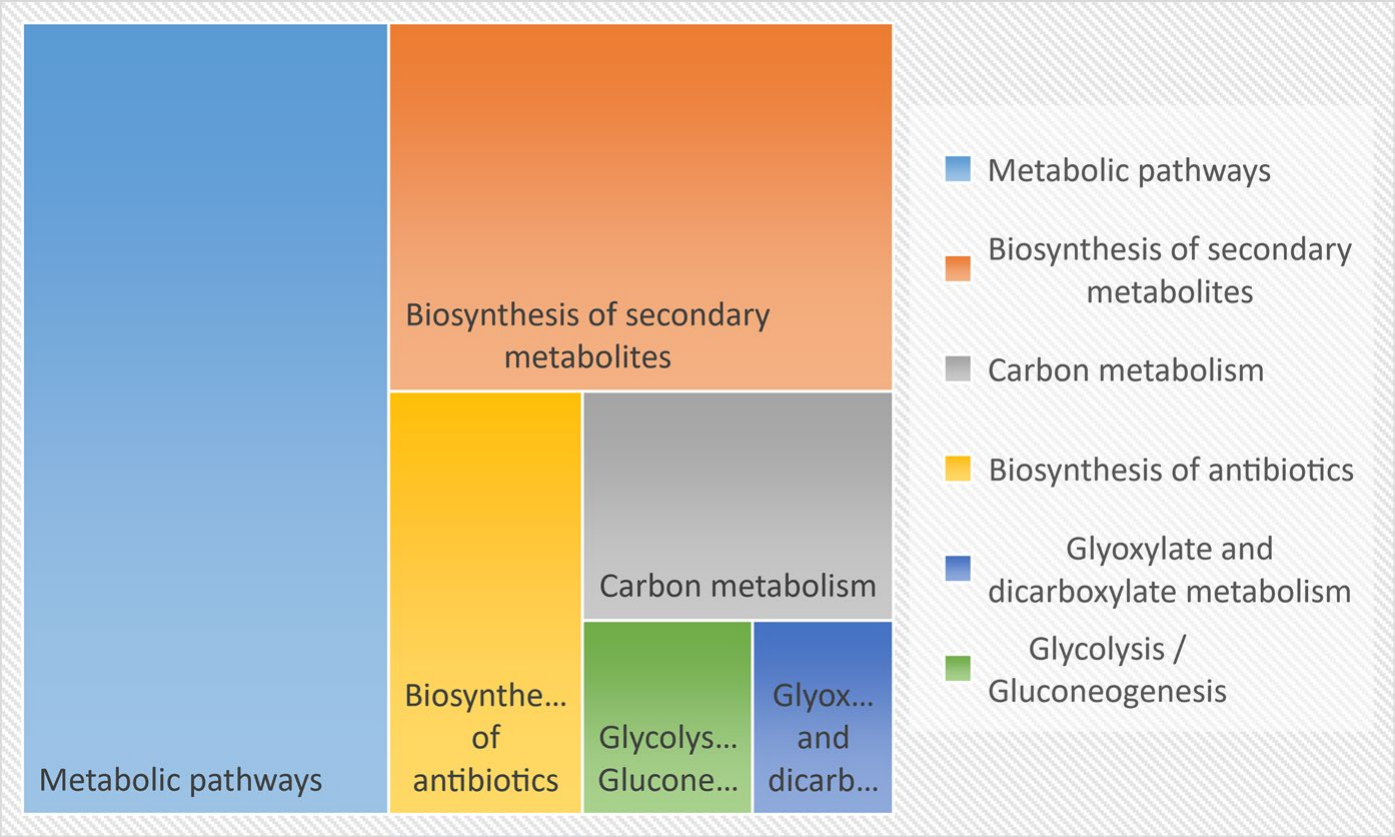
KEGG pathway analysis of the DEPs of Cu-stressed tobacco plants. KEGG (Kyoto Encyclopedia of Genes and Genomes) pathway analysis of all identified DEPs was conducted using KOBAS 3.0 (http://kobas.cbi.pku.edu.cn/help.do)

KOBAS 3.0 software was employed to analyze DEPs involved KEGG pathways. We found that all 180 DEPs were significantly enriched in six plant biological processes (*P* < 0.05) (Fig. 2). The most abundant DEP-enriched pathways were “Metabolic pathways” (53, 31.6%), “Biosynthesis of secondary metabolites” (34, 20.2%) and “Biosynthesis of antibiotics” (15, 8.9%).

### Response of antioxidative proteins to Cu stress in tobacco seedlings

Antioxidative proteins play essential roles in the response of plants to biotic and abiotic stresses. Under Cu stress, there were six antioxidative proteins that were significantly upregulated compared with the control (Table 1). Among them, three were peroxidase proteins, one catalase protein, one ascorbate peroxidase protein, and one glutathione S-transferase-like protein. The fold changes of these six proteins ranged from 1.58- to 3.16-fold. However, there were also three peroxidase proteins were also downregulated, with fold changes ranging from 0.13- to 0.46-fold. These results strongly suggest the possible involvement of antioxidative proteins in response to Cu stress in tobacco seedlings.

**Table 1:** List of antioxidative proteins significantly differentially expressed in response to Cu stress (*p* < 0.05)

Accession No	Annotation	% Cov (95)	Peptides (95%)	Fold Change	*p* value
P25819-1	Catalase 2	50.40	61	3.16	0.0001
Q9SZE7-1	Peroxidase	41.60	29	1.82	4.00E−163
Q9LVL2-1	Peroxidase	35.70	7	1.67	6.00E−42
Q42578-1	Peroxidase	47.20	118	1.63	1.00E−152
Q1PER6-1	Ascorbate peroxidase	56.40	26	1.72	2.00E−158
Q9SR36-1	Glutathione S-transferase-like protein	17.30	4	1.58	4.00E−75
Q96510-1	Peroxidase superfamily protein	50.90	18	0.46	0.036
Q96520-1	Peroxidase 4	55.80	75	0.33	0.0062
Q9SZE7-1	Peroxidase 5	24.60	9	0.13	0.0001

### Protein to protein interaction network construction

**Fig. 3:**
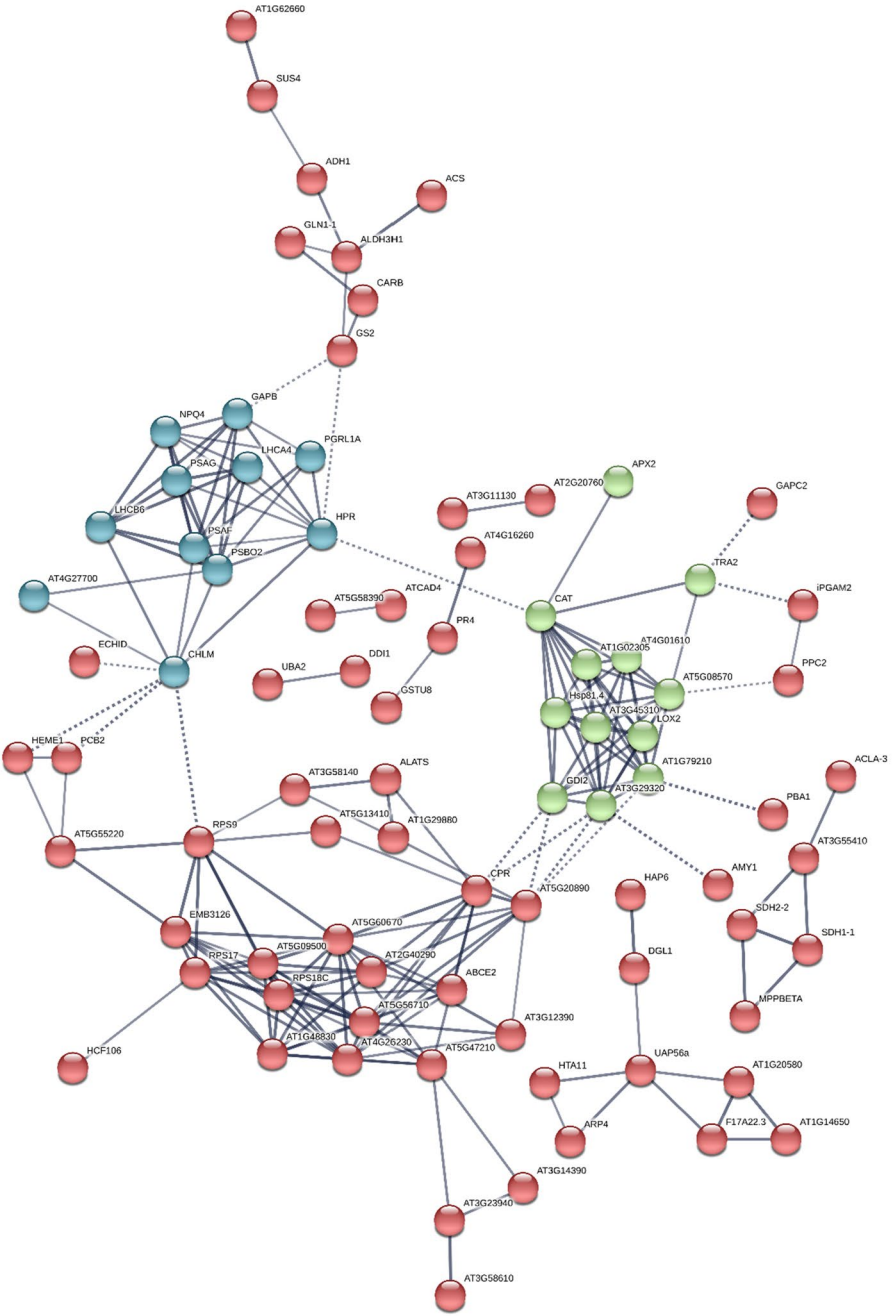
Protein–protein interaction network of the DEPs in response to Cu stress. A total of 170 DEPs were used for the construction of this network via STRING software. When running this software, the minimum required interaction score was set to high confidence (0.70), and the k-means clustering was set to 3. These DEPs can be classified into three groups. Group I (Red circle), Group II (green circle), and Group III (dark cyan) circle. A solid line indicates an interaction, and dotted line indicates a possible interaction. Line thickness indicates the strength of the data support. More information on the proteins presented in this figure can be found in Supplementary Table S3

STRING (http://string.embl.de/) software was used for the construction of a protein–protein interaction network between the 180 DEPs. The results showed that this network was composed of three groups (Fig. 3). The largest group was Group I (red circle) which consisted of 157 DEPs, followed by Group II (green circle), 12 DEPs, and Group III (dark cyan circle), 11 DEPs.

### iTRAQ results verification

**Fig. 4:**
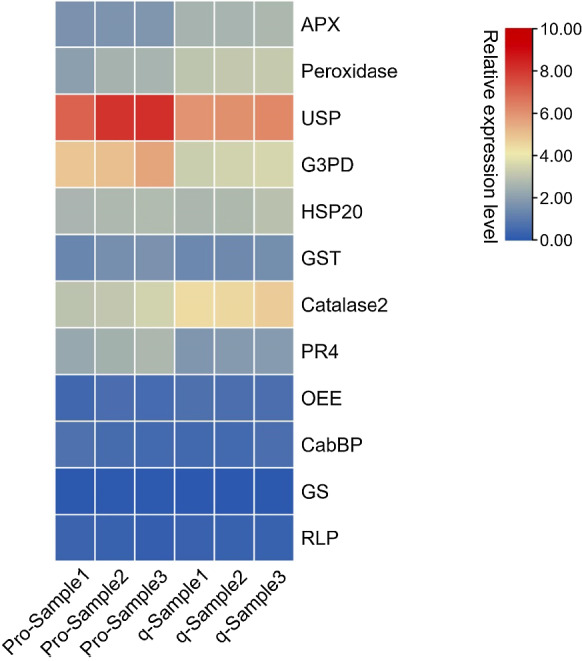
Protein abundance verification by qRT-PCR. Twelve proteins were randomly selected, and specific primers were designed according to the corresponding gene sequences. Relative gene expression level analysis was performed according to the method described in Sect. 4.5, and the 2(-Delta Delta C(T)) method (Livak and Schmittgen 2001) was used to calculate the relative gene expression level. Protein expression levels are indicated by fold change. Pro-sample 1, protein abundance of the indicated protein in sample 1; Pro-sample 2, protein abundance of the indicated protein in sample 2; Pro-sample 3, protein abundance of the indicated protein in sample 3. Gene expression levels are indicated by relative gene expression. q-sample 1, transcription level of the indicated gene in sample 1; q-sample 2, transcription level of the indicated gene in sample 2; q-sample 3, transcription level of the indicated gene in sample 3. The colour bar indicates the relative expression levels of the indicated proteins and/or genes. APX, ascorbate peroxidase; USP, universal stress protein; G3PD, glyceraldehyde-3-phosphate dehydrogenase; HSP 20, heat shock protein 20; GST, glutathione S-transferase-like protein; PR4, pathogenesis-related 4; OEE, oxygen-evolving enhancer protein 1 of photosystem II; CabBP, chlorophyll A-B binding family protein; GS, glutamine synthetase; RLP, LRR receptor-like serine_threonine-protein kinase, RLP

qRT-PCR was employed to verify the protein expression levels measured by iTRAQ. To do this, 12 randomly selected genes were recruited. The results revealed that the expression patterns of these proteins from Cu-treated tobacco plants exhibited the same tendencies as their protein abundance compared with the control (Fig. 4). This result verifies that iTRAQ technology is a reliable method to detect plant protein expression profiles.

### *Peroxidase7*-overexpressing tobacco plants exhibited increased Cu tolerance

**Fig. 5:**
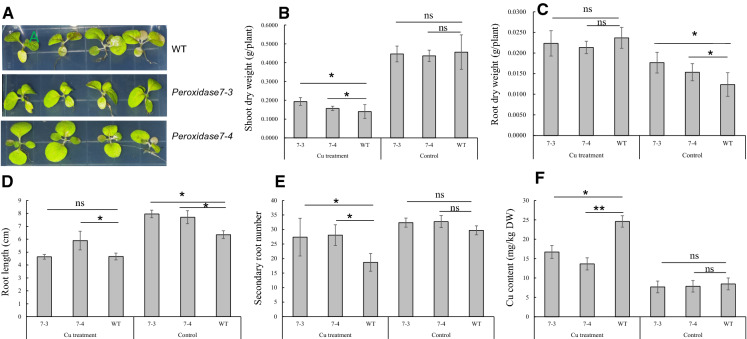
Physiological response of tobacco plants under Cu stress. **A,** Phenotypes of tobacco plants from WT and transgenic lines under 2.5 mg per ml copper sulfate treatment. **B** to **F**, Comparison of shoot dry weight, root dry weight, root length, secondary root number and Cu content between transgenic tobacco plants and wild-type plants under Cu stress. The designations 7–3 and 7–4 indicate transgenic lines. * and ** indicate significant differences between the results at *p* ≤ 0.05 and *p* ≤ 0.01, respectively. ns, indicates no significant differences between the results (*p* ≤ 0.05). Bars represent the mean ± SE (n = 3)

Several studies suggest that the ROS eliminating system plays a key role in plants in response to Cu stress (Martins et al. 2014; Leng et al. 2015; Mostofa et al. 2015; Hamed et al. 2017; Singh et al. 2020). Among the 72 upregulated proteins identified in our study, peroxidase 7 (Q9SZE7-1) expression increased by 1.82-fold. Thus, we constructed *peroxidase 7*-overexpressing tobacco lines to explore whether *peroxidase 7* overexpression could improve the tolerance of tobacco plants to Cu stress. A total of 2.5 mg per ml CuSO4 was applied to the growth medium of transgenic lines and wild type tobacco plants. Seven days after treatment, wild type tobacco plants under Cu treatment exhibited retarded growth and yellow leaves (Fig. 5A). The plants from the transgenic lines were green and no unfavorable symptoms related to Cu stress were observed. The results of the measurement of plant growth parameters showed that the shoot dry weight, root length and secondary root number of transgenic tobacco plants under Cu treatment increased by 25.0% (Fig. 5B), 12.9% (Fig. 5D) and 48.2% (Fig. 5E) respectively, and the Cu content in plant leaves decreased by 38.24% compared with the wild type (Fig. 5F).

**Fig. 6:**
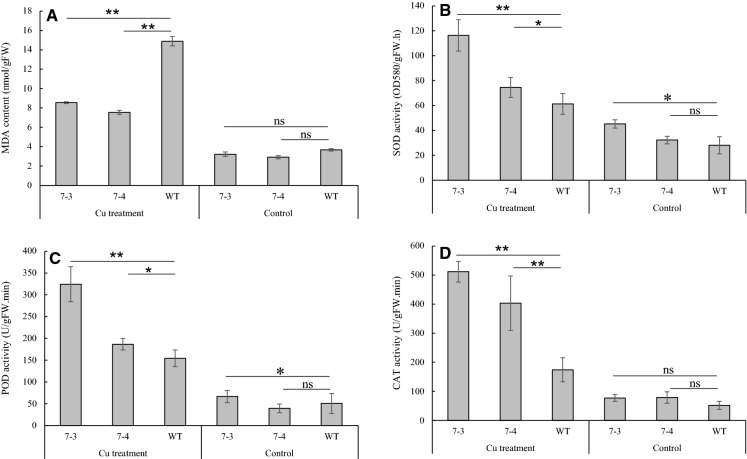
MDA content and antioxidant activity of transgenic tobacco plants under Cu stress. **A**, MDA content; **B** to **D**, SOD, POD and CAT activity, respectively. The designations 7–3 and 7–4 indicate transgenic lines. * indicates significant differences between the results (*p* ≤ 0.05). ** indicates significant differences between the results (*p* ≤ 0.01). ns, indicates no significant differences between the results (*p* ≤ 0.05). Bars represent the mean ± SE (n = 3)

To investigate the cellular damage and antioxidant enzyme response of tobacco plants under Cu stress, the MDA content and SOD, POD (type III) and CAT activities were measured in plants from transgenic lines and wild type. The results showed that excessive Cu resulted in significant increases in SOD, POD and CAT activities and a decrease in MDA concentration in the cells of transgenic tobacco plants compared to the wild type (Fig. 6).

## Discussion

### Analysis of ROS-scavenging-related DEPs

The recruitment of the members related to ROS-scavenging systems is a universal mechanism for plants to cope with unfavorable environments, such as drought, cold, salt and heavy metal stresses (Sharma and Dietz 2009; Cuypers et al. 2011; Dorneles et al. 2019; Su et al. 2019). In the present study, the expression levels of one ascorbate peroxidase, three isoforms of peroxidases, one GST (glutathione S-transferases), and one catalase increased significantly in tobacco plants treated with excessive Cu (Supplementary Table S2). It is well known that antioxidants including enzymes and nonenzyme molecules can work quite effectively in eliminating ROS generated under various abiotic stresses (Sharma and Dietz 2009). Thus, the upregulation of ROS-scavenging proteins may result in increased ROS-eliminating ability in Cu stressed tobacco plants to survive in adverse environments. Meanwhile, three isoforms of peroxidase were also downregulated in Cu treated tobacco plants, indicating the complexity of the plant mechanism in response to heavy metal stress.

### The expression of peroxidase7 significantly increased under Cu stress

It is well known that the ROS-eliminating system plays a key role in plants in response to Cu stress (Martins et al. 2014; Leng et al. 2015; Mostofa et al. 2015; Hamed et al. 2017; Singh et al. 2020). Thus, the upregulation of antioxidant enzymes was frequently observed in plants under Cu stress. For example, when treated with excessive Cu, the expression of POD exhibited a significant increase in rice plants (Song et al. 2013). In grapevine, APX, POD, CAT and GST were strongly upregulated in response to Cu stress (Leng et al. 2015). Furthermore, the upregulation of antioxidant enzymes resulted in the increased ability of *Spirodela polyrhiza* to resist Cu toxicity (Singh et al. 2020). Consistent with the previous studies, in our study, seven days after excessive Cu treatment, the abundance of peroxidase7 (Q9SZE7-1) significantly increased (by 1.82-fold) compared with the control, suggesting that peroxidase 7 may play basic roles in the tobacco response to Cu stress.

### *Peroxidase7* overexpression enhanced Cu resistance in tobacco plants

Excess heavy metal elements including Cu can inhibit chlorophyll synthesis, disturb plant photosynthesis, prevent mineral element absorption and result in the retardation of plants, and in extreme cases, plant death (Stiborova et al. 1987; Küpper et al. 2002; Malinowska et al. 2015; Malinowska et al. 2018). Therefore, the increased shoot dry weight, root length and secondary root number and decreased Cu content of transgenic tobacco plants under Cu treatment imply that *peroxidase 7* could enhance the ability of tobacco plants to resist Cu stress.

ROS are frequently observed in plants under heavy metal stress conditions and can seriously damage plant cell membranes (Sharma and Dietz 2009; Smeets et al. 2009; Cuypers et al. 2011). As a result, one of the strategies for plants to eliminate the excessive ROS is to recruit their antioxidant enzymatic-scavenging systems, such as APX, GST, SOD, POD and CAT (Cuypers et al. 2011; Thounaojam et al. 2012). Under Cu stress, the activity levels of SOD and POD increased as observed by Cuypers et al. (2011) and Thounaojam et al. (2012). These results are supported by our data (Fig. 6) indicating that increasing plant ROS scavenging ability is a universal strategy of plants employ in response to heavy metal stress. Thus, the increased activity levels of SOD, POD and CAT in *peroxidase 7* transgenic tobacco plants enhanced their ability to scavenge the excessive ROS leading to the increased tolerance of transgenic tobacco plants to Cu stress.

MDA is the result of lipid peroxidation and is an important indicator of oxidative stress caused by various stresses (Thounaojam et al. 2012). Thus, the increase in MDA content clearly exhibits the extent of cell membrane damage. Consistence with the previous studies (Naser et al. 2008; Thounaojam et al. 2012), our data showed that excessive Cu application significantly increased the MDA content of wild-type tobacco plants (Fig. 6). In contrast, transgenic plants exhibited lower MDA content under Cu stress, suggesting that *peroxidase7* overexpression increased the Cu stress tolerance of transgenic plants by ROS scavenging and lower lipid peroxidation (Fig. 6).

**Fig. 7:**
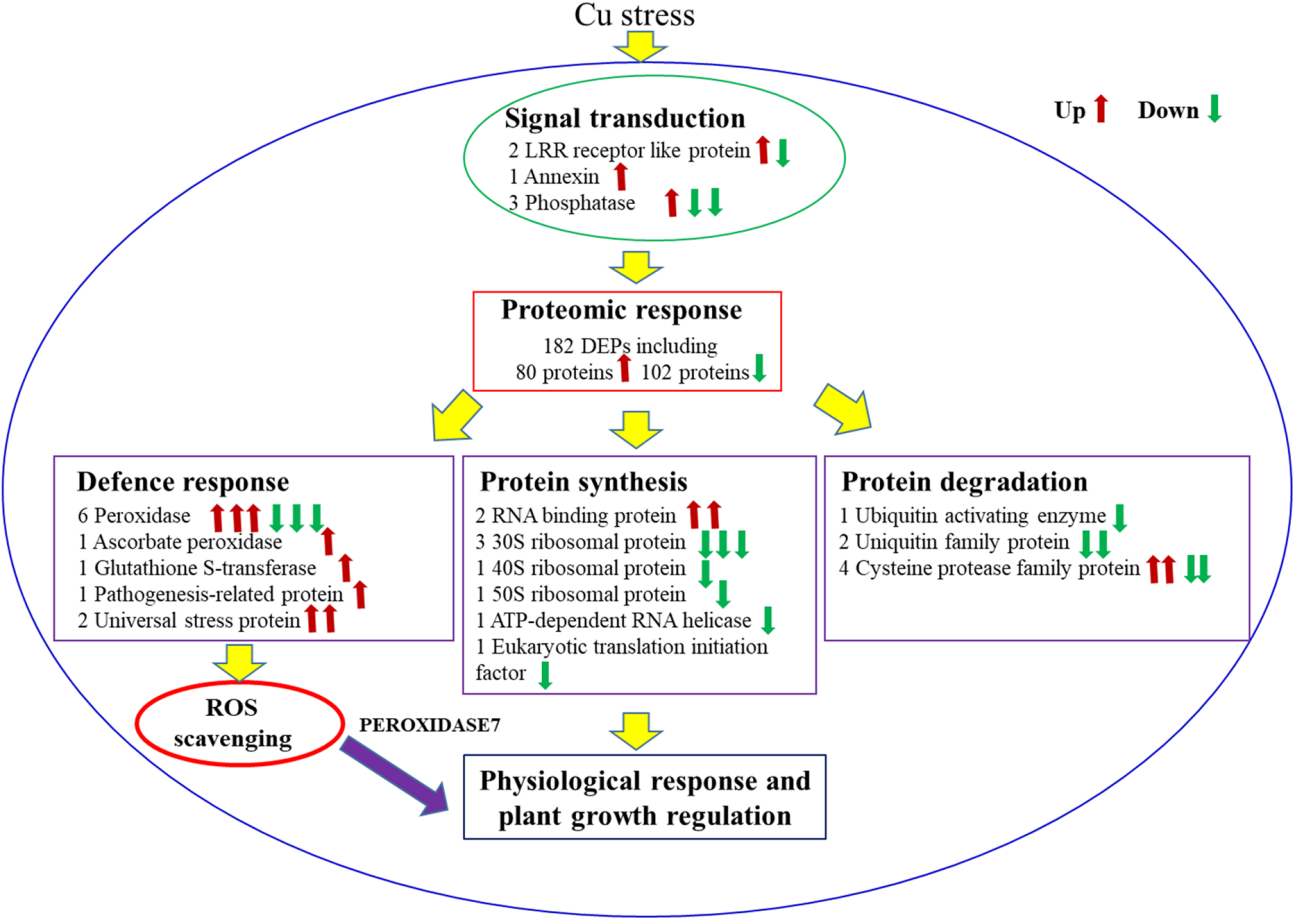
Possible working model of tobacco in response to Cu stress. The numbers before protein names indicate the numbers of these proteins differentially expressed under Cu stress. The red arrow indicates upregulation, and the green arrow indicates downregulation. (Color figure online)

In conclusion, the protein expression profile in tobacco plants was significantly altered in response to Cu stress. The physiological analysis of tobacco *peroxidase 7*-overexpressing lines clearly showed their ability to resist Cu toxicity in tobacco plants (Fig. 7). All the proteins involved worked harmoniously and rebuilt a more stable metabolic processes to ensure that the tobacco plants were able to cope with an excessive Cu environment.

## Supplementary Information

Below is the link to the electronic supplementary material.

Supplementary file1 (XLS 138 kb)

Supplementary file2 (DOC 2228 kb)

Supplementary file3 (DOC 44 kb)

Supplementary file4 (DOC 148 kb)
