# Supplementary material for: Proteome and physiological analyses reveal tobacco (Nicotiana tabacum) peroxidase 7 (POD 7) functions in responses to copper stress
Source: Transgenic Res. 2022 Jul 6;31(4-5):431–44. doi: 10.1007/s11248-022-00310-0 (PMC9489573; doi:10.1007/s11248-022-00310-0)
Supplement: Supplementary file 2 — Supplementary file2 (DOC 2228 kb) [file 11248_2022_310_MOESM2_ESM.doc]

**Supplementary Fig. S1**. Screen of positive transgenic tobacco lines. M: 2-kb ladder; WT: wild type plant; H_2_O: negative control; 7-3,7-4,7-5,7-6 and 7-11, different transgenic tobacco lines carrying tobacco *Peroxidase7* gene. Genomic DNA from different transgenic tobacco lines and wild type were extracted, and PCR was performed according to the method mentioned before.
