# Supplementary material for: Proteome and physiological analyses reveal tobacco (Nicotiana tabacum) peroxidase 7 (POD 7) functions in responses to copper stress
Source: Transgenic Res. 2022 Jul 6;31(4-5):431–44. doi: 10.1007/s11248-022-00310-0 (PMC9489573; doi:10.1007/s11248-022-00310-0)
Supplement: Supplementary file 3 — Supplementary file3 (DOC 44 kb) [file 11248_2022_310_MOESM3_ESM.doc]

| Supplementary Table S1. Primers used for PCR/qPCR analysis | | |
| --- | --- | --- |
| Gene | Primer name | Primer squence |
| NPTII | Kana-F | 5'-GCCACCTGGGATGAATGTC-3' |
|  | Kana-R | 5'-CGGTCATTTCGAACCCAGA-3' |
| Ascorbate peroxidase | ARX-qF | 5'-ATCAGAGGCAGGCTATTC-3' |
|  | ARX-qR | 5'-ATGTCAACCCAAGCAATC-3' |
| LRR receptor-like serine_threonine-protein kinase | RLP-qF | 5'-GGCATTATCAATTCCTCT-3' |
|  | RLP-qR | 5'-CTTTCTGAACCCAATCTG-3' |
| universal stress protein | USP-qF | 5'-CAGTGACAGTGGTAGGGTAT-3' |
|  | USP-qR | 5'-AGCAGTAGGGAAAGAAGC-3' |
| Glutathione transferase | GSTp-qF | 5'-CCAAGAAGCAGCCAATAA-3' |
|  | GSTp-qR | 5'-TCATAAGCAGGGAACCAG-3' |
| Glyceraldehyde-3-phosphate dehydrogenase | G3PD-qF | 5'-GAGGCTTATGAACGTCTG-3' |
|  | G3PD-qR | 5'-CCTGGCTTGTATGGAATT-3' |
| Catalase2 | CAT-qF | 5'-TGTGCCTGGGTTTATTGA-3' |
|  | CAT-qR | 5'-GTTAGCCGCCTTCACCTT-3' |
| Chlorophyll A-B binding family protein | CabBP-qF | 5'- TTTCACCGCTGCTTCCTC-3' |
|  | CabBP-qR | 5'-ACCGCACTTGTATTTGTCCT-3' |
| Oxygen-evolving enhancer protein 1 of photosystem II | OEE-qF | 5'-CTTTAGTGGCGAGTTCCT-3' |
|  | OEE-qR | 5'-GACGCAGTGTTCTTTACG-3' |
| Peroxidase | PRX-qF | 5'-AACCGTCTATACAATGAAG-3' |
|  | PRX-qR | 5'-GTGAAGAAAGTGGCTGAA-3' |
| Pathogenesis-related protein 4 | PR4-qF | 5'-TGCTTGAGGGTGACGAAC-3' |
|  | PR4-qR | 5'-TGATAGCCCACTCCATTT-3' |
| Glutamine synthetase | GS-qF | 5'-TTTGGTGCTGGTGTATGA-3' |
|  | GS-qR | 5'-AAGCGTTTAGGATAGTTGTAG-3' |
| Heat shock protein 20 | HSP-qF | 5'-CAGTGGCGTTAAGGGTCT-3' |
|  | HSP-qR | 5'-TTGGAGCAGTCAGTGGAG-3' |
| Elongation factor 1 alpha-like | EF1F | 5'-CCTACGCTCTGTATACATTAGC-3' |
|  | EF1R | 5'-GTGTTGAGTCAAATTAAGCCGC-3' |
