# Supplementary material for: Proteome and physiological analyses reveal tobacco (Nicotiana tabacum) peroxidase 7 (POD 7) functions in responses to copper stress
Source: Transgenic Res. 2022 Jul 6;31(4-5):431–44. doi: 10.1007/s11248-022-00310-0 (PMC9489573; doi:10.1007/s11248-022-00310-0)
Supplement: Supplementary file 4 — Supplementary file4 (DOC 148 kb) [file 11248_2022_310_MOESM4_ESM.doc]

| Supplementary Table S3. Information of proteins involved in protein-protein interaction network | | | | |
| --- | --- | --- | --- | --- |
| #clustering method | cluster number | cluster color | gene count | protein name |
| kmeans | 1 | Red | 147 | AT1G20580 |
| kmeans | 1 | Red | 147 | AT5G37670 |
| kmeans | 1 | Red | 147 | AT2G31670 |
| kmeans | 1 | Red | 147 | PLAT1 |
| kmeans | 1 | Red | 147 | UAP56a |
| kmeans | 1 | Red | 147 | AT5G55220 |
| kmeans | 1 | Red | 147 | F17A22.3 |
| kmeans | 1 | Red | 147 | AT2G40600 |
| kmeans | 1 | Red | 147 | PCB2 |
| kmeans | 1 | Red | 147 | AT4G25150 |
| kmeans | 1 | Red | 147 | AT2G40290 |
| kmeans | 1 | Red | 147 | MPPBETA |
| kmeans | 1 | Red | 147 | AT1G52560 |
| kmeans | 1 | Red | 147 | AT4G35160 |
| kmeans | 1 | Red | 147 | AT3G53990 |
| kmeans | 1 | Red | 147 | AT1G60000 |
| kmeans | 1 | Red | 147 | RPS17 |
| kmeans | 1 | Red | 147 | AT3G20390 |
| kmeans | 1 | Red | 147 | AT2G38870 |
| kmeans | 1 | Red | 147 | DGL1 |
| kmeans | 1 | Red | 147 | GLN1-1 |
| kmeans | 1 | Red | 147 | GSTU8 |
| kmeans | 1 | Red | 147 | PPC2 |
| kmeans | 1 | Red | 147 | UBA2 |
| kmeans | 1 | Red | 147 | RD19 |
| kmeans | 1 | Red | 147 | PCAP1 |
| kmeans | 1 | Red | 147 | AT1G14650 |
| kmeans | 1 | Red | 147 | CHIA |
| kmeans | 1 | Red | 147 | AT4G37530 |
| kmeans | 1 | Red | 147 | ACLA-3 |
| kmeans | 1 | Red | 147 | SUS4 |
| kmeans | 1 | Red | 147 | ARPN |
| kmeans | 1 | Red | 147 | AT3G14390 |
| kmeans | 1 | Red | 147 | ASA2 |
| kmeans | 1 | Red | 147 | SDH2-2 |
| kmeans | 1 | Red | 147 | AT5G56260 |
| kmeans | 1 | Red | 147 | AT2G38610 |
| kmeans | 1 | Red | 147 | iPGAM2 |
| kmeans | 1 | Red | 147 | RBCS3B |
| kmeans | 1 | Red | 147 | AT3G23600 |
| kmeans | 1 | Red | 147 | AT5G61510 |
| kmeans | 1 | Red | 147 | HAP6 |
| kmeans | 1 | Red | 147 | AT4G02320 |
| kmeans | 1 | Red | 147 | XTH7 |
| kmeans | 1 | Red | 147 | HEME1 |
| kmeans | 1 | Red | 147 | ADF4 |
| kmeans | 1 | Red | 147 | AT4G14200 |
| kmeans | 1 | Red | 147 | MGP1 |
| kmeans | 1 | Red | 147 | AT5G47210 |
| kmeans | 1 | Red | 147 | AT3G11130 |
| kmeans | 1 | Red | 147 | PR4 |
| kmeans | 1 | Red | 147 | AT3G25660 |
| kmeans | 1 | Red | 147 | LAC3 |
| kmeans | 1 | Red | 147 | ATOEP16-2 |
| kmeans | 1 | Red | 147 | GLY3 |
| kmeans | 1 | Red | 147 | AT5G13410 |
| kmeans | 1 | Red | 147 | ADH1 |
| kmeans | 1 | Red | 147 | ISU1 |
| kmeans | 1 | Red | 147 | PBA1 |
| kmeans | 1 | Red | 147 | AT1G71695 |
| kmeans | 1 | Red | 147 | HB2 |
| kmeans | 1 | Red | 147 | AT4G27270 |
| kmeans | 1 | Red | 147 | AT3G49960 |
| kmeans | 1 | Red | 147 | AT3G23940 |
| kmeans | 1 | Red | 147 | AT1G29880 |
| kmeans | 1 | Red | 147 | UREG |
| kmeans | 1 | Red | 147 | UGE1 |
| kmeans | 1 | Red | 147 | RPS9 |
| kmeans | 1 | Red | 147 | HIS1-3 |
| kmeans | 1 | Red | 147 | GLC |
| kmeans | 1 | Red | 147 | ECHID |
| kmeans | 1 | Red | 147 | CPR |
| kmeans | 1 | Red | 147 | AT1G48830 |
| kmeans | 1 | Red | 147 | AT4G31340 |
| kmeans | 1 | Red | 147 | IMPL1 |
| kmeans | 1 | Red | 147 | AT4G33720 |
| kmeans | 1 | Red | 147 | HGO |
| kmeans | 1 | Red | 147 | COR47 |
| kmeans | 1 | Red | 147 | AT3G04590 |
| kmeans | 1 | Red | 147 | ACS |
| kmeans | 1 | Red | 147 | AT3G12390 |
| kmeans | 1 | Red | 147 | GS2 |
| kmeans | 1 | Red | 147 | SPS1 |
| kmeans | 1 | Red | 147 | APS1 |
| kmeans | 1 | Red | 147 | AT2G41380 |
| kmeans | 1 | Red | 147 | AT2G44920 |
| kmeans | 1 | Red | 147 | AT2G15220 |
| kmeans | 1 | Red | 147 | AT5G09500 |
| kmeans | 1 | Red | 147 | At3g27890 |
| kmeans | 1 | Red | 147 | CARB |
| kmeans | 1 | Red | 147 | PLDALPHA1 |
| kmeans | 1 | Red | 147 | GF14 |
| kmeans | 1 | Red | 147 | PEL3 |
| kmeans | 1 | Red | 147 | AT5G60670 |
| kmeans | 1 | Red | 147 | AMY1 |
| kmeans | 1 | Red | 147 | RD21B |
| kmeans | 1 | Red | 147 | AT1G62660 |
| kmeans | 1 | Red | 147 | AT3G19320 |
| kmeans | 1 | Red | 147 | AT2G20760 |
| kmeans | 1 | Red | 147 | CRL1 |
| kmeans | 1 | Red | 147 | BGAL10 |
| kmeans | 1 | Red | 147 | DDI1 |
| kmeans | 1 | Red | 147 | ADK2 |
| kmeans | 1 | Red | 147 | RPS18C |
| kmeans | 1 | Red | 147 | GAPC2 |
| kmeans | 1 | Red | 147 | HCF106 |
| kmeans | 1 | Red | 147 | AT3G58140 |
| kmeans | 1 | Red | 147 | CUTA |
| kmeans | 1 | Red | 147 | ARP4 |
| kmeans | 1 | Red | 147 | AT5G42765 |
| kmeans | 1 | Red | 147 | LYM2 |
| kmeans | 1 | Red | 147 | AT3G62360 |
| kmeans | 1 | Red | 147 | AT5G56710 |
| kmeans | 1 | Red | 147 | AT3G58610 |
| kmeans | 1 | Red | 147 | PATL2 |
| kmeans | 1 | Red | 147 | AT3G55410 |
| kmeans | 1 | Red | 147 | AT3G05900 |
| kmeans | 1 | Red | 147 | ALATS |
| kmeans | 1 | Red | 147 | LEJ2 |
| kmeans | 1 | Red | 147 | EXLB1 |
| kmeans | 1 | Red | 147 | AT5G20890 |
| kmeans | 1 | Red | 147 | AT3G25290 |
| kmeans | 1 | Red | 147 | ABCE2 |
| kmeans | 1 | Red | 147 | TT5 |
| kmeans | 1 | Red | 147 | EFE |
| kmeans | 1 | Red | 147 | NDB1 |
| kmeans | 1 | Red | 147 | AT5G58390 |
| kmeans | 1 | Red | 147 | ATB2 |
| kmeans | 1 | Red | 147 | AT2G47710 |
| kmeans | 1 | Red | 147 | ATCAD4 |
| kmeans | 1 | Red | 147 | SDH1-1 |
| kmeans | 1 | Red | 147 | AT4G16260 |
| kmeans | 1 | Red | 147 | EMB3126 |
| kmeans | 1 | Red | 147 | AVP1 |
| kmeans | 1 | Red | 147 | PA2 |
| kmeans | 1 | Red | 147 | AT5G08540 |
| kmeans | 1 | Red | 147 | SERK2 |
| kmeans | 1 | Red | 147 | AILP1 |
| kmeans | 1 | Red | 147 | ALDH3H1 |
| kmeans | 1 | Red | 147 | AT3G05350 |
| kmeans | 1 | Red | 147 | At1g32200 |
| kmeans | 1 | Red | 147 | UXS6 |
| kmeans | 1 | Red | 147 | AT2G30620 |
| kmeans | 1 | Red | 147 | HTA11 |
| kmeans | 1 | Red | 147 | ENODL2 |
| kmeans | 1 | Red | 147 | AT4G26230 |
| kmeans | 1 | Red | 147 | TIC55-II |
| kmeans | 2 | Green | 12 | AT5G08570 |
| kmeans | 2 | Green | 12 | APX2 |
| kmeans | 2 | Green | 12 | GDI2 |
| kmeans | 2 | Green | 12 | Hsp81.4 |
| kmeans | 2 | Green | 12 | AT4G01610 |
| kmeans | 2 | Green | 12 | AT1G79210 |
| kmeans | 2 | Green | 12 | AT3G29320 |
| kmeans | 2 | Green | 12 | CAT |
| kmeans | 2 | Green | 12 | AT1G02305 |
| kmeans | 2 | Green | 12 | LOX2 |
| kmeans | 2 | Green | 12 | TRA2 |
| kmeans | 2 | Green | 12 | AT3G45310 |
| kmeans | 3 | Dark Cyan | 11 | NPQ4 |
| kmeans | 3 | Dark Cyan | 11 | HPR |
| kmeans | 3 | Dark Cyan | 11 | LHCA4 |
| kmeans | 3 | Dark Cyan | 11 | GAPB |
| kmeans | 3 | Dark Cyan | 11 | AT4G27700 |
| kmeans | 3 | Dark Cyan | 11 | PSAG |
| kmeans | 3 | Dark Cyan | 11 | PSBO2 |
| kmeans | 3 | Dark Cyan | 11 | PGRL1A |
| kmeans | 3 | Dark Cyan | 11 | CHLM |
| kmeans | 3 | Dark Cyan | 11 | PSAF |
| kmeans | 3 | Dark Cyan | 11 | LHCB6 |
